# Paternity analysis reveals significant isolation and near neighbor pollen dispersal in small *Cariniana legalis* Mart. Kuntze populations in the Brazilian Atlantic Forest

**DOI:** 10.1002/ece3.1816

**Published:** 2015-11-13

**Authors:** Evandro V. Tambarussi, David Boshier, Roland Vencovsky, Miguel L. M. Freitas, Alexandre M. Sebbenn

**Affiliations:** ^1^ Escola Superior de Agricultura “Luiz de Queiroz”Universidade de São PauloAv. Pádua Dias, 11Caixa Postal 9Piracicaba13418‐900Brazil; ^2^ Department of Plant SciencesSouth Parks RoadOxfordOX1 3RBUK; ^3^ Instituto Florestal de São PauloCP 1322São Paulo01059‐970Brazil

**Keywords:** Conservation genetics, fragmentation, inbreeding, microsatellites, population genetics, tropical tree species

## Abstract

Throughout the world, large trees are increasingly rare. *Cariniana legalis* is the tallest tree species of the Brazilian Atlantic Forest, reaching up to 60 m in height. Due to extensive deforestation of the Atlantic Forest, remnant *C. legalis* populations are small and spatially isolated, requiring the development of strategies for their conservation. For in situ and ex situ genetic conservation to be effective, it is important to understand the levels and patterns of spatial genetic structure (SGS), and gene flow. We investigated SGS and pollen flow in three small, physically isolated *C. legalis* stands using microsatellite loci. We measured, mapped, and sampled all *C. legalis* trees in the three stands: 65 trees from Ibicatu population, 22 trees from MGI, and 4 trees from MGII. We also collected and genotyped 600 seeds from Ibicatu, 250 seeds from MGI, and 200 seeds from MGII. Significant SGS was detected in Ibicatu up to 150 m, but substantial levels of external pollen flow were also detected in Ibicatu (8%), although not in MGI (0.4%) or MGII (0%). Selfing was highest in MGII (18%), the smallest group of trees, compared to MGI (6.4%) and Ibicatu (6%). In MGI and MGII, there was a strong pattern of mating among near‐neighbors. Seed collection strategies for breeding, in situ and ex situ conservation and ecological restoration, must ensure collection from seed trees located at distances greater than 350 m and from several forest fragments.

## Introduction

Throughout the last two centuries, human activities have resulted in extensive habitat fragmentation, altering the structure, distribution, and functionality of terrestrial ecosystems (Ribeiro et al. 2009). Although forest fragmentation is a global problem, it is thought to be particularly problematic in the tropics, where there is a high diversity of tree species, many of which have low population densities (Laurence et al. 2000). In tropical rain forests, tall trees with large canopies are crucial to forest survival, providing fruits, flowers and shelter for animal populations and countless other species (Laurance et al. 2000). Given the size and longevity of these trees, they are keystone species of forest ecosystems, creating optimal environmental conditions for many other plants and animals, such as insects, birds, and bats, which are the main pollinators of most tropical tree species (Chase et al. 1996). The viability of these tree populations is fundamental for biodiversity conservation in fragmented landscapes and requires knowledge of their responses to fragmentation. It is expected that reductions in forest cover and the subsequent spatial isolation of tree species populations have a negative impact on the tree species reproductive success and gene flow (Lowe et al. 2005; Manoel et al. 2012).

Forest fragmentation has serious impacts on tree genetic diversity, as well as on other organisms associated with forest ecosystems (Laurance 2012). Fragmentation modifies the movement of pollen and seeds within tree populations, disrupting ecological and evolutionary processes (Cuartas‐Hernandez et al. 2010). These disruptions may lead to lower outcrossing rates in isolated trees and small forest fragments, as well as lower genetic diversity, increased correlated mating, inbreeding, coancestry, and decreased progeny vigor (White et al. 2002; Burczyk et al. 2004; Sebbenn et al. 2011; Breed et al. 2012). High rates of selfing have been found in extremely low‐density tree populations (Lander et al. 2010; Moraes and Sebbenn 2011; Manoel et al. 2012) and small forest fragments (Tarazi et al. 2013) resulting from a change in the foraging behavior of pollinators as a result of forest fragmentation (Ghazoul 2005). As trees are predominantly outcrossing (Ward et al. 2005), they can accumulate deleterious recessive alleles, creating a high genetic load with an associated potential for inbreeding depression (Lowe et al. 2005; Breed et al. 2012). When population size declines, genetic diversity may be lost and studies of tropical trees have found signs of decreased genetic diversity in fragmented populations (Manoel et al. 2012). Identifying drastic population decreases remains an important issue because it can increase the spatial genetic structure (SGS) in fragmented populations. Increases in SGS result in an increases in mating between related individuals and local genetic drift as a result of the reduced number of reproductive individuals (Dick et al. 2008). However, long‐distance pollen dispersal may guard against these negative genetic consequences of fragmentation (White et al. 2002; Lander et al. 2010), promoting high levels of genetic diversity, larger effective population sizes, and low genetic differentiation among populations (Fortuna et al. 2008; Cuartas‐Hernandez et al. 2010).

Determining the influence of the movement of pollen on the effective population size or reproductive neighborhood area requires detailed analyses of the genetic and reproductive structure of populations. Such analyses include identifying the pollen donor and its distance from the mother tree (Burczyk et al. 2004), defining reproductive patterns within populations (variation in flowering phenology), as well as estimating the rate of migration into the population (Apsit et al. 2001). Furthermore, the effective population size is a critical parameter in population genetics because it influences the rate of genetic drift and inbreeding (Vencovsky and Crossa 2003).

In this study, we assess the effects of spatial isolation due to forest fragmentation on pollen flow, mating patterns and intrapopulation spatial genetic structure (SGS) in three stands of the tallest tree in the Atlantic Forest, *Cariniana legalis* Mart. O. Kuntze (Lecythidaceae). Today, the Brazilian Atlantic semideciduous rainforest is significantly fragmented and only 11–17% of the original forest cover remains (Ribeiro et al. 2009). This species is endemic to the Atlantic Forest, occurring at low densities (<1 tree/ha), and reaching 60 m in height and 4 m in diameter at breast height (dbh). Its flowers are hermaphroditic and pollinated by bees of the genus *Melipona* and *Trigona* (Prance and Mori 1979). Fruits can contain more than 10 seeds which are dispersed by gravity and anemochory (Carvalho 1994). Its wood is light and used in interior construction and for furniture as it is not resistant to decay. The species is considered endangered (IUCN 2002), with a need for strategies for in situ and ex situ conservation of the remaining populations, the development of which require an understanding of its genetic diversity, inbreeding, spatial genetic structure, mating system, and gene flow. As such, we address the following questions: (1) Is there intrapopulation spatial genetic structure? (2) Are stands that have been isolated spatially by forest fragmentation also reproductively isolated? (3) What are the patterns and distance of pollen dispersal within and between forest fragments? (4) Do larger trees have more descendants or is size not a factor in the number of offspring generated?

## Material and Methods

### Study sites

**Figure 1:**
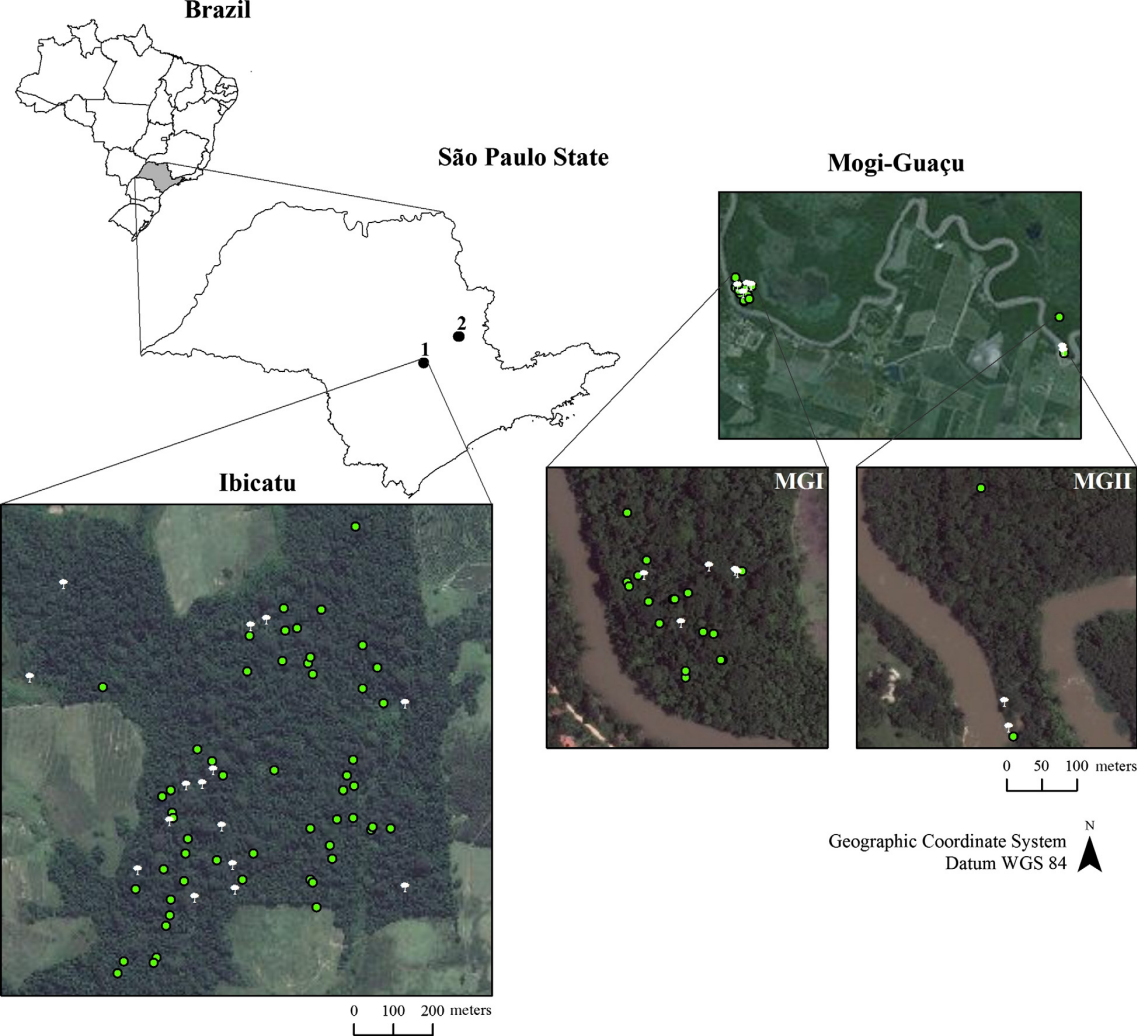
Spatial distribution of *Cariniana legalis* trees sampled in the Ibicatu State Forest and (1) Mogi‐Guaçu (2). White circles = seed trees; green circles = adults trees where seeds were not sampled.

Our study was carried out in three forest fragments near Piracicaba city, São Paulo State, Brazil (Fig. 1). One fragment is located within the Ibicatu State Forest (22°46′ S, 47°43′ W, altitude 448 to 576 masl), which is currently surrounded by agriculture (sugarcane, eucalypt, and pastures). The fragment is a remnant of a semideciduous forest which was previously subjected to several fires and selective logging. The Mata da Figueira (MGI) is a small fragment of 7.2 ha of riparian forest in the semideciduous plateau and part of the Mogi‐Guaçu Ecological Station (22°16′S 47°11′ W, mean altitude of 600 masl). Located approximately 2.9 km from MGI is a cluster of four reproductively mature *C. legalis* trees (MGII, Fig. 1). Ibicatu is isolated from other populations by at least 4 km, while MGI and MGII are 3 km apart and approximately 75 km from Ibicatu (Fig. 1). All sites have similar climates, characterized as humid and mesothermal (Köppen 1948) with variation in the mean monthly temperature between 14.3°C and 24.7°C., a mean annual temperature of 23.9°C, mean minimum 16.1°C, mean maximum 25°C. Mean annual precipitation is approximately 1320 mm, with a dry season from May to August, with 86% of the precipitation concentrated in the rainy season (September–April). The soils are yellow regosol and red podzolic “intergrades”. The Ibicatu fragment covers 72 ha with 65 *C. legalis* adult trees (0.93 trees/ha) ranging from 0.25 to 3.25 m dbh (mean 1.21 m). MGI contains 22 adults trees (3.6 trees/ha), ranging from 0.25 to 1.14 m dbh (mean 0.68 m), and MGII consists of 4 adults trees, ranging from 0.46 to 0.73 m dbh (mean 0.52 m).

### Sample size

All *C. legalis* trees in the three fragments were sampled (bark cambium samples) and mapped (using a GPS III‐Garmin, Garmin International Inc., Kansas city, MO, USA), and the dbh was measured. In August 2011, open‐pollinated fruits were harvested directly from the canopy of 15 randomly selected trees in Ibicatu, five trees in MGI, and from the only two fruiting trees in MGII. Seeds were germinated separately by fruit (mean 7.7 seeds per fruit) and seed tree with five seedlings per fruit genotyped, to give the following total genotyped: Ibicatu population 600 seed (40 seeds from eight fruits of each seed tree), MGI population 250 seeds (50 seeds from ten fruits of each seed tree), and MGII population 200 seeds (100 seeds from 20 fruits of each seed tree).

### DNA extraction and SSR amplification

Seeds were germinated in vermiculite until the cotyledons emerged, and then, DNA was extracted from the first leaf pair of 15‐ to 20‐day‐old seedlings using the method of Doyle and Doyle (1990). We used seven specific microsatellite markers specific to *C. legalis*: Cle01, Cle04, Cle05, Cle08, Cle09, Cle10, and Cle12 and selected for Mendelian inheritance, an absence of genetic linkage, and high levels of polymorphism (Tambarussi et al. 2013a,b). Details on DNA extraction, amplification, and genotyping for the seven microsatellite loci are described in Tambarussi et al. (2013a).

### Estimates of group coancestry and variance effective size

Group coancestry (Lindgren et al. 1997) for adults within fragments was estimated as the mean coancestry coefficient between all pairwise individuals (Θ), using the Nason coancestry estimator (Loiselle et al. 1995), individual fixation index (*F*), and the Spagedi 1.3 program (Hardy and Vekemans 2002). The variance effective population size was calculated as *N*_e_ = 0.5/Θ (Cockerham 1969) with the relationship between *N*_e_ and the census size of the fragment's population (*N*), calculated as *N*_e_/*N*.

### Analysis of intrapopulation spatial genetic structure

Intrapopulation spatial genetic structure (SGS) was investigated for adults in each fragment using also the estimate of coancestry coefficient between pairwise individuals (*θ*_*ij*_) as described in Loiselle et al. (1995), implemented in the Spagedi 1.3 program (Hardy and Vekemans 2002). To visualize SGS in the Ibicatu population, mean values were calculated for pairs of individuals within seven distance classes (0–100, 100–200, 200–300, 300–400, 400–500, 500–600, and 600–700 m). The number of pairs in each of these nine classes was 132, 257, 273, 353, 361, 298, and 188, respectively. To test whether the mean values were significantly different from zero, a confidence interval of 95% probability was calculated for each value in each distance class using 1000 permutations per individual between different distance classes and from which the correlogram was constructed. However, due to the fact that the number of trees in MGI (22) and MGII was very small, we only plotted the pairwise coancestry coefficient as a function of pairwise distance between the pairwise individuals. In MGI, a linear regression analysis (Sokal and Rohlf 1995) was used to determine the pairwise coancestry coefficient and was associated with pairwise distance between individuals. All these analyses were also carried out using also Spagedi 1.3 program (Hardy and Vekemans 2002).

### Paternity analysis

Contemporary pollen flow was carried out using a categorical paternity analysis, implemented in the Cervus 3.0 program (Marshall et al. 1998; Kalinowski et al. 2007). Paternity analysis for Ibicatu, MGI, and MGII was conducted using the genotypes of all adult trees (65, 22, and 4 trees, respectively), seed trees (15, 5, and 2 seed trees, respectively), and seeds (600, 250, and 200 seeds, respectively) from each stand. To determine the likely pollen donors for seeds, all adult trees in each population were used as putative pollen donor candidates. Due to the proximity of the MGI and MGII stands (<2.9 km), we grouped all 26 adult trees from both stands (22 and 4 individuals, respectively) as candidate pollen donors. In the paternity analysis, the most likely pollen donor was determined using the reference allele frequencies calculated in the adult populations as suggested by Meagher and Thompson (1987). Paternity of each seed was determined based on the ∆ statistic (Marshall et al. 1998), defined as the difference between the “LOD score” of the first most likely father candidate and the “LOD score” of the second most likely candidate. Significance was determined with the paternity tests simulated by Cervus program. The ∆ cryptic was determined based on a confidence level of 80%, as suggested by Marshall et al. (1998), using 10 000 repetitions, 0.01 as the ratio of genotyping errors, 70% as the proportion of pollen donors sampled within each population (given the high degree of isolation of the populations). The minimum number of loci necessary to determine the paternity of a seed was fixed at six. If a seed had no potential pollen donor from within the population, this seed was considered as having received the pollen from outside the population (pollen immigration). We also considered the possibility of one single mismatch in the seed‐tree‐seed trio and putative pollen donor and the possibility of self‐pollination. The selfing rate (*s*) was estimated as the proportion of seeds identified as having the same seed tree as pollen donor (*n*_*s*_) in relation to the total number of sampled seeds (*n*) *s* = *n*_*s*_/*n*. The standard error of mean selfing was estimated assuming a binomial distribution, as $SE\left(s\right)=\sqrt{s\left(1-s\right)/m_{st}}$, where *m*_*st*_ is the number of sampled seed trees in each fragment (Slavov et al. 2005). To estimate the rate of mating among relatives (*t*_*r*_), we calculated the pairwise coancestry coefficient (*θ*_*ij*_) among seed trees and assigned pollen donors by paternity analysis, using also the Spagedi 1.3 program (Hardy and Vekemans 2002). Values of *θ*_*ij*_ lower than expected between half‐sibs (0.125) were assumed as zero (unrelated). The rate of mating among relatives (*t*_*r*_) was calculated as follows: *t*_*r*_ = *n*_*r*_/*n*, where *n*_*r*_ is the number of seeds originated from mating among relatives. The pollen immigration rate (*m*) was calculated as the proportion of seeds for which a pollen donor candidate was not found within the stand (*n*_*i*_) relative to total number of sampled seeds (*n*) within the stand, *m* = *n*_*i*_/*n*, (Burczyk et al. 2004). As all sampled trees were genotyped and their spatial position known (*x* and *y* coordinates), the seeds assigned to a pollen donor were used to determine the minimum, maximum, mean and median pollen dispersal distance, as well as the standard deviation of pollen dispersal. Pollen dispersal distance (*D*) was calculated as the Euclidean distance between two points $D=\sqrt{{\left(x_{i}-x_{j}\right)}^{2}+{\left(y_{i}-y_{j}\right)}^{2}}$, where *x*_*i*_ and *x*_*j*_ are the spatial *x* coordinates of seed tree *i* and putative pollen donor *j*, assigned by paternity analysis, and *y*_*i*_ and *y*_*j*_ are the spatial *y* coordinates of seed tree *i* and pollen donor *j*, respectively). To investigate whether reproductive success was a function of the distance between trees, we compared the frequency distribution of pollen dispersal with the frequency distribution of distance between all trees using the Kolmogorov–Smirnov test (Sokal and Rohlf 1995). To investigate whether pollen donor mating success was a function of distance from the seed trees, we compared the frequency distribution of effective pollinating with the frequency distribution of the distances among the assigned pollen donors and the seed trees, using a linear regression analysis (Sokal and Rohlf 1995). The effective pollination neighbor area (*A*_ep_) was calculated by assuming a circular area around a seed tree, *A*_ep_ = 2*πσ* ^2^ (Levin 1988), where $\sigma_{p}^{2}$is the axial pollen dispersal variance. It is important to note that the parameter *A*_ep_ corresponds to the circular area in which 63% of pollen donors that crossed with a seed tree are expected to be located (Levin 1988). The circular pollination radius was estimated as $r_{\mathrm{ep}}=\sqrt{A_{\mathrm{ep}}/3.1415}$ (Austerlitz and Smouse 2001). Cryptic gene flow (*C*_GF_), or the probability of finding a compatible paternal candidate of a seed within a stand when the real father is located outside of the stand, was calculated from the probability of detecting immigrant pollen grains (*d*) in paternity analysis, given a local population of candidate pollen donors: *C*_GF_ = 1 − *d* (Slavov 2004). The parameter *d* is estimated as $d=1-\sum_{i=1}^{t}h_{i}$, (i = 1,2,…,t) using the Pollen Flow program (Slavov 2004), where *h*_*i*_ is the frequency of local pollen grain *i* in the background population, or the idealized population in which all reproductive trees could pollinate any of the sampled seed trees of the studied fragments (Slavov 2004), and *t* is the total number of distinct local pollen grains (Slavov et al. 2005). Thus, the pollen immigration rate (*m*), estimated from Cervus 3.0 program, was corrected to an unbiased pollen immigration rate, calculated using the probability of detecting immigrant pollen grains (*d*) in paternity analysis, given a local population of candidate pollen donors: *m* = *b*/*d* (Slavov 2004), (*b*) is the detected pollen immigration. The standard error of pollen immigration [*SE*(*m*)] was estimated as $SE\left(m\right)=m\sqrt{\left(1-b)/(bn\right)}$ (Slavov et al. 2005), where *n* is the number of seeds sampled from each seed tree. We also calculated the number of pollen donors that mated with each seed tree (*N*_ep_) and from this parameter the paternity correlation within progenies, *r*_*p*_ = 2/*N*_ep_ (Ritland 1989).

### Male fertility

A linear regression analysis (Sokal and Rohlf 1995) was used to determine the male fertility or if pollen donor fertility (i.e., capacity to sire offspring) was associated with dbh.

## Results

### Intrapopulation spatial genetic structure and effective population size

**Figure 2:**
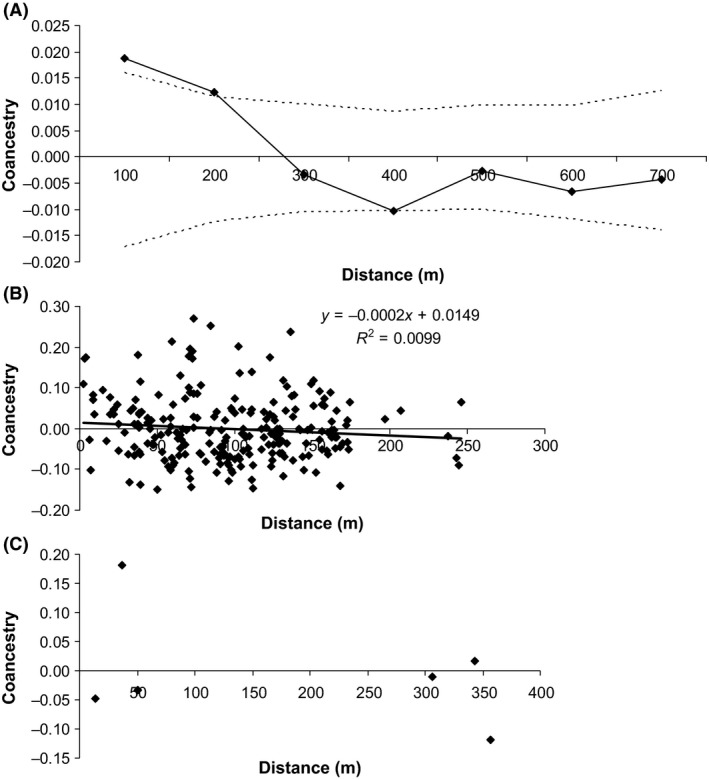
Correlograms of average coancestry coefficients (*θ*_*ij*_) of *Cariniana legalis* adult trees from Ibicatu (A), MGI (B), and MGII (C). In (A) the solid line represents the average *θ*_*ij*_ value and the dashed lines represents the two‐tailed 95% confidence interval of the average *θ*_*ij*_ distribution calculated by 1000 permutations of spatial distance among pairwise adult trees.

The coefficient of coancestry decreases with increased distance between trees in all three populations (Fig. 2). However, significant spatial genetic structure was found only in the Ibicatu population up to 150 m, suggesting that trees within this distance of each other are related. Of the three populations, the mean group coancestry of adult trees within populations was highest in the smallest population, MGII (Table 1). Thus, the effective population size was lower than the number of individuals (*N* > *N*_e_).

**Table 1:** Census number, group coancestry, effective population size, and relationship between the effective size and census number (*N*_e_/*N*) in three *Cariniana legalis* populations

Parameter	Ibicatu	MGI	MGII
Census number: *N*	65	22	4
Group coancestry: Θ	0.00747	0.02245	0.12441
Effective population size: *N*_e_	32.5	10.9	2.3
*N*_e_/*N*	0.50	0.49	0.57

### Gene flow and pollen dispersal

Of the 600 seeds sampled from the Ibicatu population, 250 from MGI and 200 from MGII population, a putative paternal parent was assigned to 553 (92.2%), 249 (99.6%), and all 200 seeds (100%), respectively (Table 2). This suggests a pollen immigration rate (*b*) from trees located outside of the fragments of only 7.8% for Ibicatu (range among seed trees 0–15%), 0.4% for MGI (range from 0% to 2%), and 0% for MGII (Table 3). In MGI, the single seed without an identified pollen donor within MGI was assigned a pollen donor from MGII. However, the probability that an immigrant pollen grain (*d*) has a detectable genotype (detected probability) from within the forest fragment ranged among seed trees in Ibicatu from 0.978 to 0.994 (mean of 0.985), in MGI from 0.995 to 0.997 (mean of 0.997), and in MGII, *d* was 0.997 for both seed trees. This resulted in an unbiased mean pollen immigration ($\hat{m}=\hat{b}/\hat{d}$) of 8%, 0.4%, and 0%, for Ibicatu, MGI, and MGII, respectively. Mean cryptic gene flow (*C*_GF_), estimated using the Pollen Flow program, was very low (0.015, 0.004, and 0.003 into Ibicatu, MGI, and MGII, respectively), indicating that cryptic gene flow is not biasing pollen flow estimates.

**Table 2:** Results of the analysis of pollen dispersal for the sampled *Cariniana legalis* populations

Population	*n*	Pollen flow	Assigned	Selfing	Mean ± SD	Median	Min–max	*A*_ep_	*r*_ep_
Ibicatu	600	47 (7.8%)	553 (92.2%)	36 (6.0%)	352 ± 244 m	341 m	19–922 m	37 ha	345 m
MGI	250	1 (0.4%)	249 (99.6%)	16 (6.4%)	63 ± 194 m	35 m	4–154 m^A^	1.44 ha^A^	68 m^A^
MGII	200	0 (0.0%)	200 (100.0%)	36 (18.0%)	130 ± 135 m	37'm	14–344 m	11 ha	191 m

*n* is the sample size; SD is the standard deviation; Min–max are the minimum and maximum distance; *A*_ep_ is the effective pollination neighbor area; and *r*_ep_ is the effective radius of pollination area. ^A^ was calculated excluding the single seed pollinated by a tree of MGII population. This unique mating event represents a pollen dispersal distance of 2929 m.

**Table 3:** Results of paternity analysis by seed tree for three *Cariniana legalis* forest fragments. *n* is the sample size; *b* is the detected pollen immigration; *d* is the probability that an immigrant pollen grain has a detectable genotype (detected probability) inside the stand; *m* is the unbiased pollen immigration rate; *SE*(*m*) is the standard error of pollen immigration; *C*_GF_ is the unbiased cryptic pollen flow; *s* is the selfing rate; *t*_*r*_ is the rate of mating among relatives; *δ* is the pollen dispersal distance estimated without selfing; *A*_ep_ and *r*_ep_ are the effective pollination and radius of neighborhood area, respectively; *N*_ep_ is the effective number of pollen donors; *r*_*p*_ is the paternity correlation; Θ and *N*_e_ are the coancestry coefficient and variance effective size within progenies, respectively; and SD is the standard deviation

Seed tree	*n*	*b*	*d*	*m* = *b*/*a*	*SE*(*m*)	*C*_GF_	*s*	*t*_*r*_	*δ* (mean ± SD) (m)	*A*_ep_ (ha)	*r*_ep_ (m)	*N*_ep_	*r*_*p*_	Θ	*N*_e_
IB‐04	40	0	0.981	0	0	0.019	0.15	0.80	60 ± 81	4.2	115	2	0.50	0.238	2.04
IB‐06	40	0.2	0.984	0.203	0.064	0.016	0.12	0.40	183 ± 233	39.1	353	7	0.14	0.188	2.53
IB‐16	40	0	0.978	0	0	0.022	0	0.30	436 ± 224	30.3	311	10	0.10	0.144	3.28
IB‐22	40	0.125	0.982	0.127	0.053	0.018	0.02	0.25	546 ± 162	16.8	231	10	0.10	0.143	3.27
IB‐23	40	0.025	0.987	0.025	0.025	0.013	0	0.43	686 ± 179	21.2	260	15	0.07	0.137	3.41
IB‐27	40	0	0.991	0	0	0.009	0	0.40	354 ± 208	23.7	275	15	0.07	0.132	3.54
IB‐28	40	0.05	0.984	0.051	0.035	0.016	0.05	0.33	419 ± 175	21.0	259	12	0.08	0.145	3.23
IB‐29	40	0.05	0.994	0.050	0.035	0.006	0	0.10	309 ± 200	20.7	257	18	0.06	0.129	3.61
IB‐30	40	0.1	0.983	0.102	0.048	0.017	0.05	0.45	235 ± 192	13.0	204	11	0.09	0.156	3.02
IB‐36	40	0.075	0.985	0.076	0.042	0.015	0	0.28	263 ± 152	14.0	211	14	0.07	0.132	3.51
IB‐41	40	0.05	0.983	0.051	0.035	0.017	0.17	0.25	224 ± 89	5.3	130	8	0.13	0.183	2.62
IB‐49	40	0.15	0.985	0.152	0.057	0.015	0	0	297 ± 206	29.5	307	9	0.11	0.145	3.21
IB‐61	40	0.1	0.988	0.101	0.048	0.012	0.07	0.15	324 ± 218	32.5	322	9	0.11	0.157	3.01
IB‐67	40	0.125	0.985	0.127	0.053	0.015	0.15	0	551 ± 264	51.3	404	8	0.13	0.177	2.69
IB‐70	40	0.125	0.986	0.127	0.053	0.014	0.05	0.30	245 ± 174	17.2	234	3	0.33	0.185	2.58
Mean		0.078	0.985	0.080	0.037	0.015	0.06	0.30	342.1	22.7	258.2	10.1	0.10	0.159	3.04
MGI‐1	50	0.02	0.995	0.020	0.020	0.005	0	0.28	15 ± 13	0.1	19	3	0.33	0.286	1.72
MGI‐2	50	0	0.996	0.	0	0.004	0.10	0.20	141 ± 437	120.1	618	7	0.14	0.163	2.94
MGI‐3	50	0	0.996	0	0	0.004	0	0	31 ± 6	0.1	8	3	0.33	0.309	1.60
MGI‐4	50	0	0.997	0	0	0.003	0.14	0	112 ± 44	1.2	62	5	0.20	0.175	2.76
MGI‐5	50	0	0.996	0	0	0.004	0	0	58 ± 50	1.6	70	10	0.10	0.138	3.44
Mean		0.004	0.996	0.004	0.004	0.004	0.05	0.10	71.4	24.6	155.4	5.6	0.18	0.214	2.49
MGII‐6	100	0	0.997	0	0	0.003	0.10	0.34	145 ± 128	10.2	180	3	0.33	0.172	2.85
MGII‐7	100	0	0.997	0	0	0.003	0.27	0.32	104 ± 137	11.8	194	3	0.33	0.209	2.36
Mean		0	0.997	0	0	0.003	0.18	0.33	124.5	11.0	187	3.0	0.33	0.191	2.61

In Ibicatu, of the seeds with an assigned father (Table 2), we identified the same individual as both seed tree and pollen donor for 36 seeds, representing a selfing rate of 6.0%. In the smaller forest fragments, 16 seeds from MGI (6.4%) and 36 (18%) seeds from MGII had the same individual as both seed tree and pollen donor. Of the 65 sampled reproductive trees in Ibicatu, 56 (86%) fathered at least one seed (ranging from 1 to 45 seeds). Of the 22 and 4 reproductive trees in MGI and MGII, 19 (86%) and 4 (100%) fathered at least one seed (ranging in MGI from 1 to 37 seeds and in MGII from 42 to 60 seeds).

### Distance and patterns of pollen dispersal

**Figure 3:**
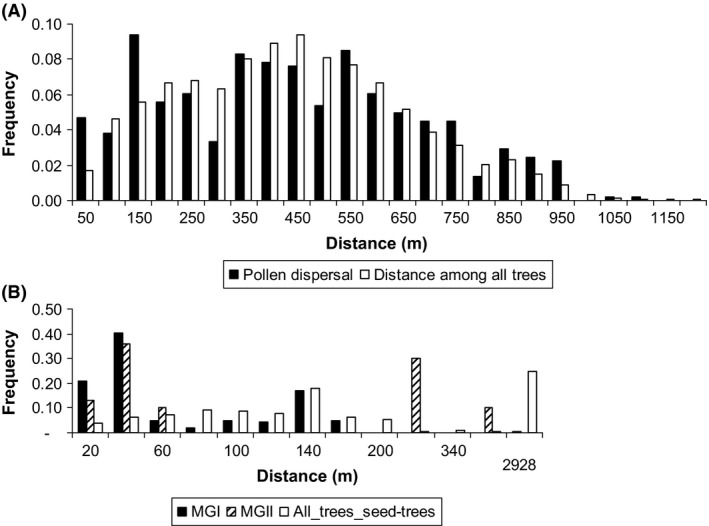
Pollen dispersal distance determined by paternity analysis in progenies and distance among all adult trees of *Cariniana legalis* in Ibicatu (A) and MGI and MGII (B) populations.

For the seeds with a putative pollen donor identified from within the same fragment, excluding selfing, the pollen dispersal distance reach in Ibicatu 922 m, 154 m in MGI, and 334 m in MGII (Table 2, Fig. 3). However, the median pollen dispersal distance was lower than mean pollen dispersal distance, indicating a pattern of isolation by distance. The single occurrence of pollen immigration into MGI from MGII represents a pollen dispersal distance of 2929 m. The mean effective pollination neighbor area (*A*_ep_) was estimated as 37 ha in Ibicatu, 1.44 ha in MGI (23 ha, considering the observed unique pollen immigrant), and 11 ha in MGII, with a mean effective neighbor pollination radius (*r*_ep_) of: 345 m in Ibicatu; 68 m in MGI (or 274 m considering the single pollen immigration event); and 191 m in MGII. Comparing the frequency curves of effective pollen dispersal and distance between all reproductive trees using the Kolmogorov–Smirnov test, we observed significant differences in Ibicatu (*D* = 0.18, *P* < 0.001), MGI (*D* = 0.461, *P* < 0.001), and MGII (*D* = 0.511, *P* < 0.001), suggesting a nonrandom distribution of pollination distance in the three fragments (Fig. 3). Moreover, there was a significant association between the number of seeds fertilized by pollen donors and the distance between paternal and maternal trees in Ibicatu (*R* ^2^ = 0.40, df = 17, *P* < 0.01) and MGI (*R* ^2^ = 0.35, df = 11, *P* < 0.05), but not in MGII (*R* ^2^ = 0.001, df = 2, *P* > 0.05).

### Male fertility

A linear regression analysis was used to determine whether pollen donor fertility (i.e., capacity to sire offspring) was associated with dbh. We found a significant association in the Ibicatu (*R* ^2^ = 0.17, df = 54, *P* < 0.01) and MGII (*R* ^2^ = 0.99, df = 2, *P* < 0.01) stands, indicating that the number of seeds produced by pollen donors is strongly associated with tree size (dbh), although no association was found for MGI (*R* ^2^ = 0.01, df = 17, *P* > 0.05).

### Individual seed trees

Pollen immigration corrected for cryptic pollen flow (*m*) was variable among seed trees of all stands (Table 3), especially in Ibicatu (ranged from 0% to 20.3%). Selfing rate ranged from 0% to 17% in Ibicatu, 0% to 14% in MGI, and 10% to 27% for the two seed trees in MGII. The pollen dispersal distance (*δ*) was higher for seed trees from Ibicatu (maximum of 686 m) than for seed trees from MGI (maximum of 145 m) and MGII (maximum of 145 m). Effective pollination neighbor area (*A*_ep_) was lower in Ibicatu (maximum of 51.3 ha) than in MGI (maximum of 120.1 ha) and MGII (maximum of 11.8 ha). The maximum radius of effective pollination neighbor area (*r*_ep_) was 404 m in Ibicatu, 618 m in MGI, and 194 m in MGII. The mean effective number of pollen donors (*N*_ep_) per seed tree was higher in Ibicatu (10.1) than MGI (5.6) and MGII (3.0), resulting in mean paternity correlations (*r*_*p*_) of 0.10, 0.18, and 0.33, respectively. Due to selfing and correlated mating, the mean coancestry coefficient within progeny (Θ) was lower in Ibicatu (0.159) than in MGI (0.214) and MGII (0.191), resulting in a higher mean variance effective size (*N*_e_) within progeny in Ibicatu (3.04) than MGI (2.49) and MGII (2.61).

## Discussion

### Intrapopulation spatial genetic structure

Significant spatial genetic structure (SGS) was found only in the Ibicatu stand up to 150 m (Fig. 1). However, coancestry values between pairwise individuals (*θ*_*ij*_) up to a distance of 150 m showed many high values: 29% of values ranged from 0.062 to 0.486 (distances ranging from 146 to 179 m), which are in the range between first cousins (*θ*_*ij*_ = 0.0625) and self‐sibs (*θ*_*ij*_ = 0.5). Even at greater distances (180–1184 m), there were high levels of coancestry, with 23% of values (ranging from 0.062 to 0.406 at distances of 180–1123 m). Thus, although coancestry was not always structured spatially, many trees within stands are highly related, even at greater distances. SGS in natural populations can be caused by short‐distance seed and pollen dispersal (Bacles et al. 2006). Our analysis of pollen dispersal for the Ibicatu population showed that short‐distance dispersal could be contributing to SGS as approximately 30% of mating occurred between trees located within 150 m (Fig. 3). As many trees are related, there is a substantial degree of mating among related trees (ranging from zero to 0.8, mean of 0.3). Due to inbreeding and the presence of a high number of related individuals in all stands, the estimated effective population size was lower than the number of individuals (*N*) within stands resulting in a *N*_e_/*N* relationship that is lower than unity (minimum of 0.49). Without pollen flow, the populations have reduced evolutionary potential because even with high levels of genetic diversity, the high levels of coancestry within stands may lead to inbreeding due to mating among relatives, resulting in inbreeding depression.

### Mating patterns and pollen flow

Paternity analyses suggest that selfing may be higher in stands with fewer *C. legalis* trees. We detected higher rates of selfing in MGII (18%), the smallest stand, as compared to MGI (6.4%) and Ibicatu (6%). The habitat of the *C. legalis*, the Atlantic Forest was strongly reduced and fragmented in least centuries (Ribeiro et al. 2009) and due the natural low densities population occurrence of the species (<1 tree/ha), a low number of reproductive individuals actually remaining the stands. Thus, our results suggest the small remaining populations of the species may present highest selfing. Other studies of tropical tree species have observed similar patterns (e.g., Lander et al. 2010; Kamm et al. 2011; Moraes and Sebbenn 2011; Manoel et al. 2012; Tarazi et al. 2013), as did a study of the insect pollinated, Mediterranean climate tree *Gomortega keule,* which found a selfing rate of 56% in isolated trees, 30% in small stands, and 22% in large stands (Lander et al. 2010).

Our results show that the spatially isolated Ibicatu and MGI stands are not reproductively isolated (Tables 2 and 3), although the four isolated trees (MGII) appear to be reproductively isolated. Furthermore, rates of pollen flow into Ibicatu and MGI were low (8% and 0.4%, respectively). In the region surrounding the Ibicatu population, we found some reproductive size *C. legalis* trees in two areas, at distances of about 2.5 km and 5.4 km. Although there were other forest fragments in the region, these closest trees are the most likely sources of the 8% pollen immigration, demonstrating the efficiency of bees in facilitating gene flow in tree species. In the MGI stand, the closest conspecifics were the four trees of the MGII stand, at a distance of 2.9 km, one of which was the pollen donor of the single MGI seed detected as sired by immigrant pollen. Although there were some *C. legalis* trees located about 4.9 km from MGI and about 2.2 km from MGII, our sampling of the 2011 reproductive event suggests that neither stand received pollen from these trees.

Clearly, if pollen immigration rates are consistent across all reproductive events within Ibicatu and MGI, pollen flow may be enough to maintain or increase genetic diversity and effective population size, despite some inbreeding and SGS. Nevertheless, it is very important to note that we investigated effective pollen dispersal of open‐pollinated seeds collected from the canopy of seed trees, not the realized pollen dispersal, as measured through regeneration. Differences between the seed dispersal phase and the seedling or juvenile phases may be significant, although inbred material is often selected against in these phases. In fact, although many trees were producing large numbers of fruits in both the Ibicatu and MGI populations, we did not find substantial regeneration in either fragment, suggesting that the rate of seedling establishment is low or at best episodic. We do not have enough data to investigate the cause of this phenomenon. However, inbreeding depression is unlikely to be a substantial cause as the majority of seeds in all fragments were produced by outcrossing.

### Distance and patterns of pollen dispersal

Pollen dispersal within Ibicatu reached longer distances (maximum 922 m, mean of 352 m) than in MGI (maximum 154 m, mean of 63 m) and MGII (maximum 344 m, mean of 130 m). The one event detected of pollen immigration from MGII to MGI (2929 m) shows that pollen dispersal distance is greater than the maximum internal values for each stand. Leal et al. (2014) studding pollen dispersal distance in a logged population of *C. legalis* report a mean of 334 m and the maximum of 1055 m, supporting our results showing long pollen dispersal distance in the species. The effective pollination neighbor area (*A*_ep_) represents a circular area around a seed tree containing 63% of pollen donors that mated with the seed tree (Levin 1988) and was variable among seed trees (range 0.1–51.3 ha). These estimates produce mean radii of effective neighbor area (*r*_ep_) of 345, 68, and 191 m, in Ibicatu, MGI, and MGII, respectively. Thus, seed trees received pollen from a large area and over long distances, much higher than the results detected for other bee‐pollinated neotropical tree species, *Copaifera langdsdorffii* (*A*_ep_ seed trees range 0.05–4.15 ha, mean 0.68 ha; Manoel et al. 2012) and *Cordia alliodora* (*A*_ep_ seed trees range 3.25–7.28 ha; Boshier et al. 1995).

Although the type of pollinator partly determines the distances over which pollen moves (Dick et al. 2008; Mitchell et al. 2009), pollen dispersal is also affected by tree population density and isolation between stands. At low densities, trees are more widely dispersed and pollinators must fly longer distances than in high density populations. Our results support the view that differences in pollen dispersal distance occurred as a result of differences in population densities in Ibicatu (0.93 trees/ha) and MGI (3.6 trees/ha).

The patterns of pollen dispersal were also different among the studied fragments. In Ibicatu, mean (352 m) and median (341 m) dispersal distances were similar, while in MGI and MGII, the median dispersal distance (both at 37 m) was substantially lower than the mean dispersal distance (63 and 130 m, respectively), indicating the tendency of mating among near neighbor trees in these fragments (Fig. 3). Furthermore, there was a significant association between the number of seeds fertilized by pollen donors and the distance between paternal and maternal trees both in Ibicatu and MGI. The Kolmogorov–Smirnov test, which compares the frequency curve of effective pollen dispersal with the frequency curve of distances between all reproductive trees, suggested nonrandom mating in all three forest fragments (Fig. 3).

Bees may show fidelity to very small fragments even though a fragment may be several kilometers from the hive (Monzon et al. 2004). Thus, isolation of fragments will be mediated by pollinator foraging range and behavior (Ghazoul 2005; Mitchell et al. 2009; Wang et al. 2010), with a tendency to forage in small areas or among a few trees of a stand. Such behavior was observed on *Stachys officinalis* by bumblebees, which visited a high number of inflorescences, but tended to remain longer in smaller stands (Goverde et al. 2002). Gérard et al. (2006) noted that a nonrandom spatial assortment of mating events among conspecifics is mediated by pollinators. Thus, pollen donors do not distribute pollen evenly among seed trees, but rather among a nonrandom subset of available seed trees (Fortuna et al. 2008).

Ghazoul (2005) conducting a review of pollen and seed dispersal among widely dispersed plants found that for 123 species across 59 families, approximately 68% of the studied species were pollinated by some kind of bee. In tropical forests, bees are the predominant pollination vector facilitating gene flow across forest fragments, with 25% of 40 tropical tree species in which pollen flow was studied, pollinated by bees (Dick et al. 2008). Pollen dispersal distances in nine bee‐pollinated species, in continuous forests, have reportedly reached 3.5 km, while for four species occurring in fragmented populations, pollen dispersal reached 3.1 km (Degen and Sebbenn 2014).

### Male fertility

An important aspect of reproductive biology is understanding male fertility and whether larger trees are more successful pollinators than smaller trees. Male mating success has been shown to increase with proximity, flower intensity, and tree size (Burczyk et al. 1996; Klein et al. 2008; Mori et al. 2009). Linear regression showed a significant association between dbh and the number of fathered seeds in both the Ibicatu and MGII populations and between the number of seeds fertilized by pollen donors and the distance between the paternal and maternal trees in Ibicatu and MGI. Positive correlation between dbh size and male fertility has also been found in *Pinus attenuata* (Burczyk et al. 1996) and *Prunus ssiori* (Mori et al. 2009). However, Oddou‐Muratorio et al. (2005) concluded that for the bee‐pollinated, temperate tree species, *Sorbus torminalis*, the spatial position of a pollen donor in relation to a seed tree is just as important a determinant of male fecundity. The effect of dbh on fertility can be related to the development of large crowns with high flower production and easy access for bees and other insects (Plowright and Galen 1985). The large flowering crowns of emergent canopy trees are conspicuous and pollination vectors may use them as landmarks to help orient their foraging over long distances (Plowright and Galen 1985).

## Conclusions

Our results show that only the Ibicatu stand has a substantial effective population size, due to the greater number of trees. Although the studied stands were spatially isolated, in the single reproductive event evaluated, there was substantial pollen flow into the Ibicatu population, which is essential in maintaining genetic diversity, effective population size, and reducing inbreeding and SGS. However, pollen flow into the smaller stands MGI and MGII was very low, with the smallest MGII stand also having higher rate of selfing. Thus, seeds in very small stands may have higher levels of inbreeding than seeds from larger populations. Our results can be used to inform seed collection strategies for breeding, in situ and ex situ conservation and ecological restoration. For these purposes, it is very important to ensure genetic diversity by collecting seeds from: (1) nonrelated seed trees; (2) seed trees that do not mate with each other; (3) seed trees that do not receive an overlapping pollen pool. Our results from the Ibicatu stand indicate that to adhere to the first requirement (1), seed trees must be separated by at least 150 m to avoid collecting seeds from related seed trees. However, to adhere to requirements (2) and (3), the mean pollen dispersal distance (352 m) and radius of effective pollinator neighbor area (*r*_ep_ = 345 m) suggest that seed trees must be separated by at least 352 m. Clearly, even in the largest stand, Ibicatu, these requirements would result in the collection of seeds from a small number of seed trees. Thus, we must collect seeds from other stands to reach a minimum number of 45 seed trees, as suggested by Sebbenn (2006).

## Conflict of Interest

None declared.
